# Seizures and Blurred Vision as Initial Presentation of Intracerebral Schwannoma: A Rare Tumor of the Brain

**DOI:** 10.1155/2019/8158950

**Published:** 2019-02-10

**Authors:** Mohammad Alayyaf, Nour Taher Nasir

**Affiliations:** ^1^Consultant Histopathologist and Nephropathologist, Head of Anatomic Pathology Department, Riyadh Regional Lab & Blood Bank, Saudi Arabia; ^2^Almaarefa College for Science and Technology, Collage of Medicine, Riyadh, Saudi Arabia

## Abstract

Schwannomas are the most common tumor of peripheral nerves which are arising from Schwann cells and are benign in their nature. Intracranial schwannoma accounts for between 5 and 8% of intracranial tumors, whereas intracerebral schwannoma, a rare disease, accounts for <1% of intracranial schwannomas. Intracerebral schwannoma has no specific clinical manifestation, and it is not classified by age. Here, we are reporting a case of an 18-year-old male who presented with attacks of seizures. MR imaging studies were done and showed right parieto-occipital cortical and subcortical mass lesion with intense enhancement and significant vasogenic oedema with mass effect on the subjacent sulci. The tumor was surgically removed through a right occipital craniotomy. Histological findings confirmed the diagnosis of schwannoma.

## 1. Introduction

Schwannoma represents approximately 8% of all intracranial tumors predominantly arising from the vestibular portion of the VIII cranial nerve. [1] Intraparenchymal schwannomas of the brain and spinal cord are very rare, accounting for <1% of intracranial schwannoma and less than 100 cases have been reported, 4 of them in the parieto-occipital area. [2–4] We report a case of right parieto-occipital schwannoma in an 18-year-old boy who presented with seizure.

## 2. Case Report

**Figure 1:**
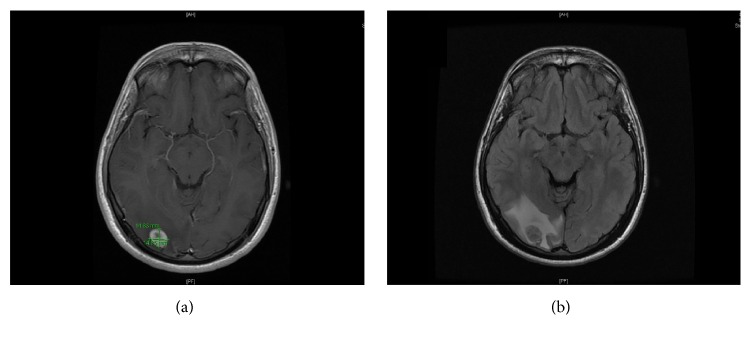
Brain MRI with contrast showed right parieto-occipital cortical and subcortical mass lesion measuring about 1.5 x 1.5 cm.

The patient is an 18-year-old boy not known to have any medical illness previously, who was referred to the outpatient department from another hospital after 3 months history of seizures. Seizures started with blurring of vision and proceed to right sided head deviation and tonic posturing of the right upper limp which progress to generalized tonic-clonic seizures. The episodes are followed by loss of consciousness and postictal suboccipital and frontal tension headache. There was no history of fever, loss of weight, trauma, or any sensory/motor neurodificit. Family history was unremarkable, with negative past surgical history. All general and local physical examinations were done and all were within normal range. There were no physical findings or family history in favour of neurofibromatosis. Brain MRI with contrast was done and showed right parieto-occipital cortical and subcortical mass lesion measuring about 1.5 x 1.5 cm that has a low signal intensity on T1 and intermediate signal intensity on T2 and FLAIR with intense enhancement postgadolinium administration mainly peripherally with few small susceptibility artefact on T2 associated with significant vasogenic oedema and mass effect on the adjacent sulci (Figure 1).

**Figure 2:**
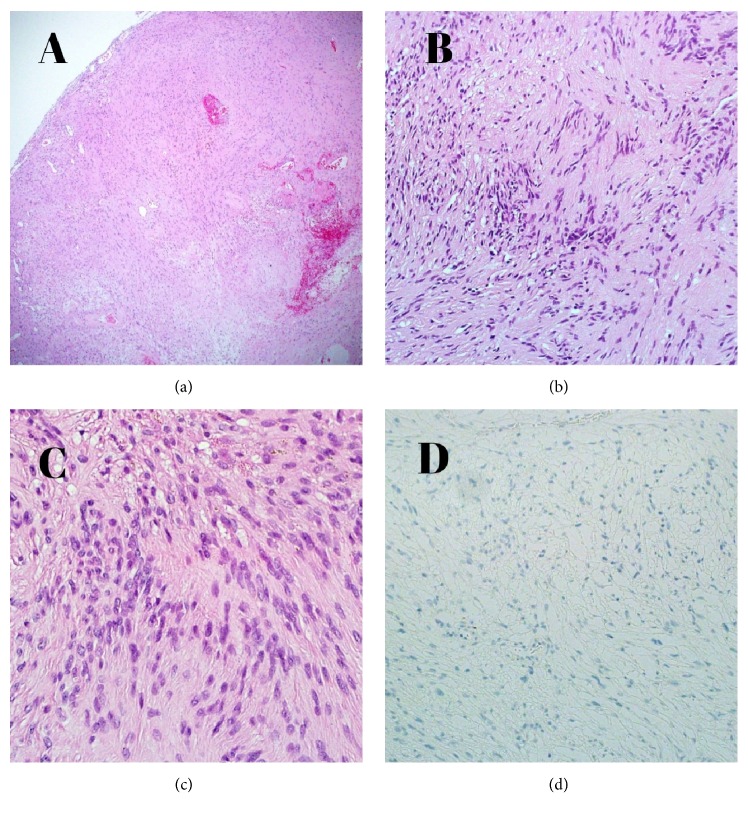
(a) Microscopic examination of the tissue under a low power with H&E staining. Under a moderate power (b) and a high power (c) magnification, areas of nuclear palisading of bland looking spindle cells with dense cellular area alternating with loosely textured myxoid area, consistent with Antoni type A and Antoni Type, B respectively. (d) Epithelial membrane antigen protein staining was negative.

The radiological impression with the above description was most likely representing granulomatous infection (TB) or metastasis. Although a preoperative diagnosis could not be clearly established, the tumor was surgically removed through a right occipital craniotomy. The dura was incised and small area of discoloration was noted. Cortical dissection was done, with multiple pieces for frozen section, which was diagnosed later as suggestive of schwannoma, differential diagnosis meningioma. The tumor was encountered 2mm in subcortical area, which was firm, fibrous in content, yellowish in colour, and resembling meningioma. Postoperatively, the patient did not have any new neurological deficit, and he was discharged 1 day postoperatively in stable condition to be seen and followed up in the clinic after 2 months. He was kept on phenytoin 100 mg orally three times a day and Paracetamol 650 mg tablets 4 hourly PRN. Microscopic examination of the tissue showed areas of nuclear palisading of bland looking spindle cells with dens cellular area alternating with loosely textured myxoid area, consistent with Antoni type A and Antoni Type B, respectively (Figure 2).

Immunohistochemical study was diffusely positive for S100 protein and negative for EMA, confirming the diagnosis of schwannoma (WHO grade1).

## 3. Discussion

Schwannomas arising within the central nervous system unrelated to major cranial nerves are extremely rare. [5] Most of reported cases were located in central hemispheres; only 4 cases were reported to be in the parieto-occipital area. [6] There are still debates on histogenesis of intra-axial schwannoma and various theories have been suggested. These include the presence of Schwann cells along the perivascular nerve plexus, as well as differentiation of multipotential mesenchymal elements in central nervous system into Schwann cells. [7, 8] One hypothesis is that these tumors represent a developmental abnormality. The strongest evidence for this argument is the young age of patients at presentation. Scant molecular or histological evidence, however, supports this hypothesis. [1] The differential diagnosis of intracerebral schwannoma includes several neoplasms that may occur in children or young adults. These include pilocytic astrocytoma, pleomorphic xanthoastrocytoma, ganglioglioma, and meningioma. The specimen should be examined using immunohistochemical studies in order to reach the correct diagnosis. [6, 9] The reported neuro-adiological characteristics are thought to be of high frequency of calcification, cystic formation, and oedema which is surrounding the tumor [10]. Radiologically, in our case there were no cystic components and no calcification or vasculitis.

## 4. Conclusion

Intracerebral schwannoma is an extremely rare benign neoplasm. It is usually located superficially or adjacent to ventricle. These tumors cannot be preoperatively differentiated from other parenchymal tumors. Surgical excision is curative and the long-term prognosis is excellent.

## Data Availability

The data that support the findings of this study are available on request from the corresponding author, Dr. Alayyaf. The data are not publicly available due as they contain information that could compromise the privacy of research participants.

## List of Abbreviations

TB: Tuberculosis
PRN: Pro Re Nata (*Latin*): When necessary.
